# Vaccine Potentiation by Combination Adjuvants 

**DOI:** 10.3390/vaccines2020297

**Published:** 2014-04-14

**Authors:** Benoît Levast, Sunita Awate, Lorne Babiuk, George Mutwiri, Volker Gerdts, Sylvia van Drunen Littel-van den Hurk

**Affiliations:** 1 VIDO-Intervac, University of Saskatchewan, 120 Veterinary Road, Saskatoon, SK S7N 5E3, Canada; E-Mails: benoit.levast@mail.mcgill.ca (B.L.); sua784@mail.usask.ca (S.A.); george.mutwiri@usask.ca (G.M.); volker.gerdts@usask.ca (V.G.); 2 3-7 University Hall, University of Alberta, Edmonton, AB T6G 2J9, Canada; E-Mail: lbabiuk@ualberta.ca; 3 School of Public Health, University of Saskatchewan, 107 Wiggins Road, Saskatoon, SK S7N 5E5, Canada; 4 Veterinary Microbiology, University of Saskatchewan, 52 Campus Drive, Saskatoon, SK S7N 5B4, Canada; 5 Microbiology and Immunology, University of Saskatchewan, 107 Wiggins Road, Saskatoon, SK S7N 5E5, Canada

**Keywords:** review, adjuvants, combinations, human clinical trials

## Abstract

Adjuvants are crucial components of vaccines. They significantly improve vaccine efficacy by modulating, enhancing, or extending the immune response and at the same time reducing the amount of antigen needed. In contrast to previously licensed adjuvants, current successful adjuvant formulations often consist of several molecules, that when combined, act synergistically by activating a variety of immune mechanisms. These “combination adjuvants” are already registered with several vaccines, both in humans and animals, and novel combination adjuvants are in the pipeline. With improved knowledge of the type of immune responses needed to successfully induce disease protection by vaccination, combination adjuvants are particularly suited to not only enhance, but also direct the immune responses desired to be either Th1-, Th2- or Th17-biased. Indeed, in view of the variety of disease and population targets for vaccine development, a panel of adjuvants will be needed to address different disease targets and populations. Here, we will review well-known and new combination adjuvants already licensed or currently in development—including ISCOMs, liposomes, Adjuvant Systems Montanides, and triple adjuvant combinations—and summarize their performance in preclinical and clinical trials. Several of these combination adjuvants are promising having promoted improved and balanced immune responses.

## 1. Introduction

Adjuvants are crucial components of vaccines, both for human and animal applications. Adjuvants were initially developed empirically by co-formulating vaccine antigens with a variety of molecules including oils, salts, and carbons. Our growing understanding of the immune system, however, and in particular the innate immune system, has enabled us to develop adjuvants according to a more rational and focused approach rather than through “trial and error.” Indeed, adjuvant research has become an integral part of vaccine development. It combines a variety of disciplines, including chemistry, biochemistry, molecular biology, and immunology. Many novel adjuvant technologies have been developed or are in the pipeline for future vaccine candidates. Such novel technologies include combination adjuvants, which consist of more than one adjuvant component and which often act synergistically by stimulating and activating a variety of cells and immune mechanisms. Here, we will review several of the best-known combination adjuvants, including liposomes, ISCOMs, montanides, nanoemulsions and Adjuvant Systems, and summarize their performance in preclinical and clinical trials.

## 2. Liposomes

### Cationic Liposomes

Cationic liposomes have been studied for many years as delivery vehicles/adjuvants. Liposomes protect antigens from degradation, deliver them to antigen presenting cells (APCs), and can be used for mucosal delivery. Many liposomes mediate retention of the vaccine at the site of delivery, and some have immunostimulatory properties, although frequently other compounds such as toll-like receptor (TLR) ligands need to be incorporated for optimal adjuvanticity. Cationic liposomes may contain the following lipids: dimethyldioctadecylammonium (DDA), 3-(*N*-[*N*',*N*'-dimethylaminoethane]-carbamoyl) cholesterol (DC-Chol), 1,2-dioleoyl-3-trimethylammonium-propane (DOTAP), *N*-(1-[2,3-dioleyloxy] propyl)-*N*,*N*,*N*-trimethylammonium (DO^TM^A), octadecenoyloxy (ethyl-2-heptadecenyl-3-hydroxyethyl) imidazolinium (DOTIM), *N*-palmitoyl-d-erythrospingosyl-1-*O*-carbamoyl spermine (CCS), 1,2-dioleyl-sn-glycero-3-ethylphosphocoline (DOEPC) and 3-tetradecylamino-tert-butyl-*N*-tetradecylpropion-amidine (diC14-amidine) (reviewed by [1]). The mechanism of action, while not completely understood yet, is dependent on the nature of the lipids in the formulation, and thus varies widely between different liposomes. One common characteristic related to the cationic charge is the enhanced uptake of DNA or protein by target cells [2]. One of the major drawbacks of liposomes as vaccine delivery vehicles has been their instability. While numerous animal trials have been reported, only recently have liposome-based vaccine formulations moved into human clinical trials. This has become possible by the incorporation of helper lipids stabilizing the liposome formulations [2]. 

DDA has been used as adjuvant in animal trials since 1966 [3], either alone or in combination, and was tested in humans in 1970 and 1973 in combination with tetanus toxoid and alum [4,5]. However, although the vaccine was safe and induced antibody responses, these were not considered robust enough. Consequently, DDA has been combined with other compounds including Emulsigen^®^, monophosphoryl lipid A (MPL), trehalose dibehenate (TDB), monomycoloyl glycerol (MMG) and polyI:C. We combined DDA with Emulsigen and found robust immune responses in cattle [6]. One of the most promising combinations is DDA with TBD (cationic adjuvant formulation [CAF01]). TDB not only stabilized the liposomes, but also significantly enhanced T-cell responses to *Mycobacterum bovis* Ag85B-ESAT-6 in mice, in particular T cells producing IFN-γ and IL-17; this correlated with an increase in IgG2 levels, while IgG1 levels remained the same [7]. The immune responses induced by Ag85B-ESAT-6 formulated with CAF01were long-lived and protective [8]. In addition to mediating depot formation of the vaccine formulation, CAF01 appears to promote influx/activation of DCs into the injection site [9]. Interestingly, in a recent report, the antibody and CD8^+^ IFN-γ responses induced by small unilamellar DDA/TDB liposomes were higher than those elicited by multilamellar DDA/TDB liposomes; however, addition of TLR3 or TLR9 ligand enhanced the immune responses, in particular CD4^+^ and CD8^+^ T cells, induced by the multilamellar ones, though this was not found for smaller liposomes [10]. CAF01 has been or is being tested in several phase I clinical trials, one against tuberculosis in combination with Ag85B-ESAT-6 (ClinicalTrails.gov identifier NCT00922363) and two with the HIV peptide cocktail AFO-18 (ClinicalTrials.gov identifier NCT01141205) (Table 1). 

MPL (a TLR4 ligand) and MMG, two of the other compounds combined with DDA, are both found in bacterial cell walls and promote Th1-biased immune responses. MPL is derived from LPS, a TLR4 ligand, and when included in an Ag85B-ESAT-6—DDA liposome formulation, it enhanced protection against tuberculosis both in mice and in cynomolgus monkeys [11,12]. Prominent inflammatory responses to DDA/MPL were observed in subcutaneously immunized mice, including high local levels of pro-inflammatory cytokines, chemokines and a pronounced influx of neutrophils, monocytes/macrophages and activated natural killer cells [13]. The antigen-specific T-cell responses induced by CAF01 (DDA/TDB), DDA/MPL and DDA/MMG in mice were all comparable. However, whereas all three compounds are immunostimulatory, TDB and MMG have the advantage over MPL of stabilizing the liposomes and are thus more promising. 

DOTIM was originally used for *in vitro* and *in vivo* gene delivery into cells [14]. However, in view of its ability to mediate uptake of DNA into endosomes [15], it was more recently combined with DNA, TLR3 or TLR9 ligand. When co-administered with antigen, the combination of DOTIM and CpG ODN promoted significantly enhanced antigen-specific T-cell responses when compared to delivery of protein with CpG ODN alone [16]. The DOTIM-based liposome in combination with cholesterol and plasmid DNA, designated JVRS-100 adjuvant, promotes pro-inflammatory responses followed by the development of Th1-type responses. Formulations with JVRS-100 have been shown to be efficacious in rodent models against several viruses including hepatitis B virus [17], influenza virus [18], herpes simplex virus-2 (HSV-2) [19], and Rift Valley fever virus [20]. This liposome formulation is also suitable for mucosal delivery as demonstrated in mice where protection from pneumonic tularemia [21] and plague [22] was induced. Currently, JVRS-100 adjuvant is being tested in phase I and phase II clinical trials with an influenza split vaccine (ClinicalTrials.gov identifier NCT00936468; ClinicalTrials.gov identifier NCT00662272) (Table 1). 

**Table 1:** Liposomes and TLR agonists: Clinical studies.

Adjuvant	Phase	Vaccine antigen	Outcomes	References.
CAF01	I	*Mycobacterium tuberculosis* Ag85B-ESAT-6	No study results posted	ClinicalTrails.gov identifier NCT00922363
CAF01	I	HIV peptide cocktail AFO-18	Induction of T-cell responses in some of the vaccinees; no significant changes in viral load or CD4^+^ T cell counts	ClinicalTrials.gov identifier NCT01141205 [23]
JVRS-100 adjuvant	I	Fluzone^®^	No study results posted	ClinicalTrials.gov identifier NCT00662272
JVRS-100 adjuvant	II	Fluzone^®^	No study results posted	ClinicalTrials.gov identifier NCT00936468

## 3. Immune Stimulating Complexes (ISCOMs)

Immune stimulating complexes (ISCOM) are ring-like structures containing cholesterol, phosphatidylcholine and saponins, mostly QuilA. ISCOMs have been tested in a variety of species, including small and large animals. The actual ISCOM matrix can directly associate with antigens or be formed first and then added to the formulation at a later time. An important advantage of ISCOMs is their excellent stability for over one year at 4 °C. While the exact mechanism of action is still unclear, ISCOMs have proven to be highly effective when delivered via mucosal surfaces. 

ISCOMs interact with dendritic cells (DCs) and can enhance cross-presentation of the incorporated antigen [24], followed by an efficient induction of both CD4^+^ and CD8^+^ antigen-specific T-cell responses [25]. ISCOM vaccines are known to induce long-lasting antibody responses, a balanced Th1/Th2 response, and induction of cytotoxic T lymphocytes [26]. ISCOMs have been used in combination with a parenterally administered H5N1 influenza vaccine in healthy adults (PANFLUVAC). In this study, 50 μg of a 3rd Generation ISCOMs was combined with various concentrations of recombinant hemagglutinin (HA). The trial is currently underway (ClinicalTrials.gov identifier NCT00868218) (Table 2). In another clinical trial, ISCOMs have been used with the melanoma vaccine NY-ESO-1 ISCOMATRIX in patients with measurable stage III or IV melanoma. Four doses of the vaccine containing 120 μg ISCOMATRIX combined with 100 μg of NY-ESO-1 protein were given to patients with advanced melanoma. The trial is currently ongoing. To improve their efficacy, ISCOMs are often used in combination with immune stimulators such as Cholera Toxin A (CTA) subunit or TLR ligands. For example, fusion proteins have been tested including the CTA1-DD, which combines the enzymatically active CTA1-subunit with a B-cell targeting moiety (DD), derived from *Staphylococcus aureus* protein A [27,28]. Intranasal immunization of mice with hemagglutinin and neuraminidase from influenza strain PR8 resulted in enhanced IgA and CD4^+^ T cells [29]. Mucosal IgA and CD4^+^ T-cell response were enhanced after intranasal administration and consequently provided 100% protection against challenge infection. The mechanism was CTA1 enzyme-dependent and designed to target B cells. Another example includes co-formulation of ISCOM and a TLR9 ligand (CpG ODN). In particular, when used in cancer vaccines, this combination resulted in regression of tumors in a pancreatic cancer mice model using tumor induction by injection of PANC02 tumor cell line transfected by OVA gene [30]. The vaccine, ISCOM+TLR9+OVA antigen, was able to induce strong immunity against the tumor and induce effective memory responses, including a high percentage of CD8^+^IFNγ^+^ T cells after re-induction of the tumor. Noteworthy, antigen and ISCOM alone or CpG ODN alone were not efficient to induce anti-tumoral immune response and protection in mice. Another example to improve ISCOMS includes tubular immunomodulatory complexes [31,32] or the association with another adjuvant such as Montanide [33]. For example, when used in combination with a recombinant protein of food and mouth disease virus (FMD) in guinea pigs, higher immune responses were found when ISCOMs and Montanide ISA 206 were used in combination. 

**Table 2:** ISCOMs and IC31^®^: Preclinical and clinical studies.

Adjuvants	Phase	Subjects	Comments	References
NY-ESO-1-ISCOMATRIX	II	Individuals with advanced melanoma	Study ongoing	ClinicalTrails.gov identifier NCT 00518206
ISCOM and PANFLUVAC	I	Healthy Adults	Study ongoing	ClinicalTrails.gov identifier NCT00868218
IC31^®^/Ag85B-ESAT-6 (TB vaccine)	Preclinical	Neonatal murine model	↓ Bacterial growth, differentiation of CD4^+^ T cells into multifunctional Th1 and Th17 cells secreting IL-2, TNF-α and IFN-γ, antigen-specific DCs expressing co-stimulatory molecules CD80, CD86 and CD40 in the DLNs	[34,35]
IC31^®^/Ag85B-ESAT-6 (TB vaccine)	Preclinical	Cynomolgus macaques	↓ Clinical infections and prevents reactivation of latent infections	[36]
IC31^®^/Ag85B-ESAT-6 (TB vaccine)	Preclinical	Cynomolgus macaques	↓ Clinical infections and prevents reactivation of latent infections	[36]
IC31^®^/Ag85B-ESAT-6 (TB vaccine)	I	Healthy mycobacterially naïve individuals	Potent and persistent antigen-specific IFN-γ^+^ T-cell responses, sustained for 2.5 years	[37]
IC31^®^/Ag85B-ESAT-6 (TB vaccine)	I	Prior TB-infected individuals	Strong antigen-specific T-cell responses, sustained for 32 weeks	[38]
IC31^®^/H1N1 (Influenza vaccine)	Preclinical	Young and aged mice	↑ HI titers, IgG2a Abs and IFN-γ producing CD4^+^ T-cell responses, sustained for 200 days after a single dose vaccination	[39]
IC31^®^/HSV-2 (Genital herpes vaccine)	Preclinical	Mice	High neutralizing HSV-specific Ab responses and splenic IFN-γ responses	[40]

## 4. Montanide

Montanide-based adjuvants have been used in both veterinary and human vaccines. These oil-based formulations have been successfully commercialized and are now available for animals in a vaccine against FMD. For human application, Montanide-based therapeutic vaccines have been developed for cancer [41]. The mechanism of action for this oil-based adjuvant includes the formation of a depot at the injection site, which enables the slow release of the antigen. Formulated antigens are concentrated and protected against degradation while phagocytosis is stimulated. 

Montanides can be improved by combining them with other adjuvants or immune modulators. An example of a Montanide-based adjuvant is the Incomplete Seppic Adjuvant (ISA™, SEPPIC Inc. Fairfield, NJ, USA), which acts as oil-emulsion adjuvant, and which has been tested in combination with a number of experimental vaccines including a malaria vaccine [42,43]. Also, Montanides and ISCOMs were tested with a recombinant protein vaccine for FMD virus using guinea pigs [33]. Interestingly, this combination showed the greatest promise in inducing early and long-term protection. Since activation of the innate immune system is one of the best ways to stimulate a systemic immune response, Montanides have been tested in combination with TLR ligands including LPS for TLR4, Poly (I:C) for TLR3, Imiquimod for TLR7 and CpG ODN for TLR9. 

Montanides have been evaluated in several clinical trials. For example, the Montanide ISA 51 was tested in combination with cancer antigens and cytokines including an evaluation of the effects of local Granulocyte-Macrophage Colony Stimulating Factors (GM-CSF) on skin DCs in melanoma patients [44,45]. Interestingly, low doses of GM-CSF did not enhance its immunogenicity [46]. Montanides and Poly (I:C) have been used for a phase I trial on 28 advanced ovarian cancer patients [47]. Using overlapping long peptides (OLP) from the human cancer-testis antigen NY-ESO-1, patients were treated with OLP alone, OLP+Montanide or OLP+Montanide+polyIC LC. No NY-ESO-1-specific antibodies or CD8^+^ T cells were detected after vaccination with OLP alone, though they were found in 46% (Ab) and 62% (TCD8^+^) patients respectively after vaccination with OLP+Montanide, and in 91% (both) patients respectively after vaccination with OLP+Montanide+Poly-ICLC. These results strongly indicate the importance of the adjuvants, especially when combined in a single formulation. In another study in melanoma patients, imiquimod was added to a combination of viral nanoparticles coated with CpG ODN and Montanide. The combination resulted in greater memory and effector CD8^+^ T cells responses [48]. Finally, CpG ODN was assessed to be efficient in cancer vaccine therapy with the same antigen NY-ESO-1 [49]. Treated patients were able to produce specific CD8^+^ T cells. Interestingly, for three different leukemia-associated antigens, no positive results were found using the CpG–Montanide combination [50]. Although only tested in very few subjects, it underlines the importance of the choice of adjuvants and antigen. Furthermore, Montanides have been tested in combination with very small size proteoliposomes (VSSP) in the treatment of prostate cancer [51]. VSSP is already included in a combination of adjuvants. However, the authors showed that association of a Gonadotropin Releasing Hormone (GnRH)-peptide with VSSP and Montanide ISA-51 was able to reduce size and weight of both testicles and prostate in a male rat model.

## 5. IC31^®^

Polycationic peptides enhance cellular uptake of proteins or bacterial DNA by cells [52]. IC30, which consists of poly-l-arginine, has been shown to efficiently transport tumor antigens to APCs [53]. IC30 adjuvant activity involves recruitment of MHC class II (+) cells at the injection site and migrating antigen-specific cells to the draining lymph nodes. Subcutaneous injection of IC30 with tumor antigen-derived peptide antigens led to antigen-specific T-cell responses, which were detectable for more than four months after injection suggesting a depot effect [54].

Based on the ability of cationic compounds to enhance uptake, IC30 was combined with CpG ODN, a TLR9 agonist. The combination of IC30 and CpG ODN induced stronger antigen-specific T cells responses, which were detectable even one year after injection [55]. Apart from IC30, various other cationic antimicrobial peptides have been developed to treat infections. The potential of an antibacterial cationic peptide KLKL_5_KLK (KLK) to induce humoral and Th2-type immune responses against co-administered antigens after prime-boost immunizations have been reported [56]. This led to development of the novel adjuvant IC31^®^, where the potential of KLK to induce Th2 responses and that of TLR9 agonist to induce Th1 responses was exploited. The novel bi-component vaccine adjuvant, IC31^®^ (Intercell AG, Vienna, Austria) consists of a vehicle based on KLK and the TLR9 agonist ODN1a. ODN1a, a single-stranded DNA-phosphodiesther, consists of repeats of the dinucleotides deoxyinosine and deoxycytosine [oligo-d(IC)13] [57]. CpG ODN was replaced by ODN1a due to potential side effects induced by CpG ODN such as production of systemic pro-inflammatory cytokines [58]. The preclinical studies with IC31^®^ in various disease models in animals have shown potent immunogenicity and protection efficacy including antigen-specific humoral and cellular responses. These encouraging results pave the way for the novel combination adjuvant IC31^®^ into clinical trials against various viral and bacterial infections (Table 2). 

The two components of IC31^®^, KLK and ODN1a, act via different mechanisms. Subcutaneous injection of fluorescent-labeled OVA, KLK, and ODN1a in mice led to depot formation at the site of injection, which was detectable up to 58 days after injection. However, co-administration of fluorescent-labeled OVA and ODN1a resulted in rapid clearance from the injection site, indicating the potential of KLK to induce a depot at the injection site [59]. IC31^®^ formed a stable complex that protected both adjuvants and antigens from degradation leading to continuous stimulation of the immune system and induction of specific immune responses. In addition, co-administration of OVA with IC31^®^ in TLR9 and MyD88 double knock-out mice completely abolished antigen-specific immune responses suggesting that the immune-stimulatory effects of IC31^®^ depends on TLR9/MyD88 signaling pathways [59]. Since cationic peptides have the potential to enhance uptake of bacterial DNA, KLK may serve as a delivery vehicle to transport ODN1a inside the murine and human DCs to interact with intracellular TLR9 receptors [60]. Further engagement of TLR9 leads to NF-κB activation via the adaptor molecule MyD88, thereby triggering the immune responses. 

Being the only professional APCs, DCs are specialized in processing and presenting antigens to naïve T cells and induce activation of antigen-specific immune responses [61]. Presentation of antigens on MHC molecules, expression of co-stimulatory molecules, and secretion of cytokines are essential for optimal activation of naïve T cells. IC31^®^ has been shown to upregulate the surface expression of MHC class I and co-stimulatory molecules CD80, CD86, CD40 and CD54 on murine bone-marrow dendritic cells (BMDCs). Further, in *in vitro* studies using OVA-pulsed BMDC, IC31^®^ induced antigen-specific CD4^+^ Th cells proliferation and differentiation of OVA-specific CD4^+^ T cells into antigen-specific Th1 cell (IFN-γ producing) and Th2 cell (IL-4 producing) types [59]. *In vivo* studies suggests involvement of plasmacytoid DCs (pDCs) in the adjuvant activity of IC31^®^, as highly activated pDCs with enhanced expression of co-stimulatory molecules were found in the draining lymph nodes of mice injected with IC31^®^. In addition, IC31^®^ induced potent cytotoxic effector T lymphocytes that killed target cells in an antigen-specific manner [59]. 

## 6. Triple Combination Adjuvant

Various combination adjuvants consisting of a variety of molecules have been developed in recent years and are currently being tested in preclinical trials. This includes both dual and triple combinations and includes polymers such as poly(lactic-co-glycolic) acid, chitosan and polyphosphazenes [62,63]. A novel combination adjuvant was developed consisting of innate defense regulator peptide (IDR), polyphosphazene (PP) and TLR ligand, either CpG ODN or PolyI:C. Innate defense regulator peptides are short peptides that stimulate both innate and adaptive immunity and by activating antigen-presenting cells link innate and adaptive immunity. Innate defense regulator peptides are involved in a variety of immune functions including innate immune activation, wound healing, cell recruitment and to some extent, direct antimicrobial control [64,65,66]. Polyphosphazenes are synthetic polymers that are used as carriers for adjuvants and vaccine antigens and that on their own have modest adjuvant activity [67,68]. However, when combined with immune stimulators such as CpG ODN or PolyIC these molecules can form complexes that become highly immunogenic. The combination adjuvant was developed through screening of hundreds of molecules and testing in human, murine and porcine cells. The most promising candidates were assessed *in vivo* in a variety of animal species including cattle, pigs, sheep, mice and cotton rats in combination with a variety of vaccine antigens. Interestingly, when co-formulated, the adjuvants induced strong expression of cytokines and chemokines—such as Gro1a, TNF-α, IL-6—in stimulated cells and displayed a strong synergistic effect when used in combination [69]. Furthermore, the adjuvant platform can be formulated into microspheres that range in size from 100 nm to 2 μm and that enhanced the mucosal immune response following intranasal immunization. Interestingly, the proper selection of innate defense regulator peptide was found to be critical as some of these peptides could actually decrease the immune response [70]. These microparticles could be lyophilized and stored at room temperature for more than a year without any loss of specific activity [71]. A synergistic effect of the TLR agonist, IDR and PP on the magnitude of the humoral, and specifically cell-mediated, immune responses was demonstrated in murine and bovine models [72,73,74]. The adjuvant platform was highly effective against a variety of infectious diseases. For example, when combined with the *Bordetella pertussis* antigens pertussis toxoid, filamentous hemagglutinin, and/or pertactin, the vaccine induced protective immune responses in both mice and pigs against lethal infection with *B. pertussis* [75]. The vaccine induced an earlier onset and longer duration than existing commercial vaccines and was effective already after a single immunization even in the presence of maternal antibodies. Furthermore, the vaccine shifted the immune response to a more balanced or Th1-type response [76,77], Similarly, when the same adjuvant combination was used with a recombinant fusion protein of respiratory syncytial virus, strong immune responses were found that provided protection against infection in mice, cotton rats and lambs [78,79], When used in combination with chlamydia antigens or influenza virus antigens, strong immune responses were detected in vaccinated animals [69,80]. 

## 7. Adjuvant Systems

Adjuvant systems^™^ (GlaxoSmithKline (GSK)) are various combinations of classical adjuvants and immunostimulators specifically designed to tailor the adaptive immune responses against specific pathogens in a target population including children, elderly and immunocompromised individuals. Various adjuvant systems have been developed and a few are in clinical trials. AS03 and AS04 have already been licensed for use in humans. AS03 was approved for H5N1 prepandemic and H1N1 pandemic influenza vaccines and AS04 for human papilloma virus (HPV) (Cervarix™) and hepatitis B virus (HBV) (Fendrix^®^). AS04 was also licensed for herpes simplex virus (HSV) but was recently terminated due to low efficacy in clinical trials [81].

### 7.1. AS04

The TLR4 agonist lipopolysaccharide (LPS) is a potent adjuvant but it is very toxic and causes septic shock. MPL is a “detoxified” derivative of LPS isolated from the Gram-negative bacterium *Salmonella Minnesota* R595 strain. While less toxic, MPL retains the immunostimulatory properties of LPS. AS04 (Adjuvant System 04), is a combination adjuvant containing MPL and aluminum hydroxide. AS04 is licensed for use in HPV and HBV vaccines. Another promising HSV vaccine, gD2/AS04, showed better immune protection and significantly reduced the viral load and viral shedding when compared to the gD2 vaccine adjuvanted with aluminum salts alone in guinea pigs [82]. In clinical trials, gD2/AS04 tested in HSV-1 and HSV-2 seronegative women showed 73% and 74% efficacy respectively against genital herpes infections [83]. However, in a randomized, double-blind phase III clinical trial conducted in 8323, 18–30 year-old, women seronegative for HSV-1 and HSV-2, the HSV-2/AS04 vaccine efficacy was only 20% and it failed to protect the women from genital disease [84].

When combined with alum, MPL still retains its ability to interact with TLR4 and activate innate immune responses. This leads to activation of NF-κB and transient production of pro-inflammatory cytokines (IL-6, TNF-α, and IFN-γ) and chemokines, such as CCL2 and CCL3 at the injection site [85]. CCL2 and CCL3 can promote recruitment of other immune cells, especially monocytes and macrophages, and activation and maturation of APCs, especially DCs at the site of injection. Increasing numbers of mature DCs migrate to the draining lymph nodes and interact with T cells to trigger the induction of antigen-specific adaptive immune responses [86]. MPL induces Th1-type immune responses by promoting IFN-γ production by antigen-specific CD4^+^ T cells, hence overcoming the shortcoming of alum, which induces only Th2-type immune responses. When combined with alum, MPL-induced stimulation of pro-inflammatory cytokines was neither improved nor inhibited by alum. However, alum did prolong the local cytokine responses induced by MPL at the injection site [85]. 

### 7.2. AS03

Since water-in-oil emulsions were too reactogenic for use in humans, GSK developed AS03, an oil-in-water emulsion. AS03 consists of dl-α-tocopherol, squalene, and polysorbate 80. Oil-in-water emulsions are less reactogenic and have better safety profiles. One such oil-in-water emulsion is MF59, which has been approved for human use in European countries. MF59 consist of squalene, polysorbate 80 and Span 85 and the only difference between MF59 and AS03 is the presence of α-tocopherol, a biodegradable and immunostimulatory oil. α-tocopherol is a form of vitamin E that is easily absorbed in human body [87]. Squalene, another component of AS03, is normally synthesized in the liver and found in various organs with highest secretion by human skin. Squalene is an essential intermediate molecule in the biosynthesis pathways of steroid hormone, cholesterol and vitamin D [88]. The last component of AS03, polysorbate 80 (polyoxy-ethylene sorbitan-20 monooleate) is added to stabilize the emulsion. It is a semi-synthetic molecule derived from naturally occurring fatty acid that is largely used as aqueous formulation stabilizer in the food and pharmaceutical industries [89]. 

Injecting antigen and AS03 72 h apart or in different limbs resulted in reduced influenza-specific antibody titers suggesting that AS03 adjuvant activity depended on the spatial and temporal co-localization with the antigen [90]. AS03 induced production of NF-ĸB, and subsequently cytokines and chemokines, including CCL2, CCL3, IL-6, CSF3 and CXCL1 in muscle and the draining lymph nodes of mice after injection [90]. Similar to alum and MF59, DCs were not directly activated by AS03. Antigen-loaded DCs, monocytes and granulocytes were found to migrate to the draining lymph nodes after injection of AS03. H5N1 split-virion A/Vietnam/1194/2004 influenza vaccine with AS03(A) resulted in increased production of polyfunctional H5N1-specific CD4^+^ T cells in volunteers aged 18–60 years [91]. An increase in antigen-specific CD4^+^ T cells might be responsible for persistence of increased antibody responses and induction of memory B cell responses by AS03. 

### 7.3. AS02

AS02 is a combination of oil-in-water emulsion with MPL and QS21 (MPLA+QS21+o/w emulsion) and was initially developed for malaria vaccines. QS21 has been shown to induce antigen-specific antibody, cell-mediated and CTL responses, including development of immunological memory in nonhuman primates [92,93,94]. AS02 was used for development of the RTS,S candidate malaria vaccine, which includes the circumsporozoite protein (CSP) as a vaccine antigen. RTS,S consists of a hybrid protein that includes a portion of the CSP fused to the hepatitis B virus surface antigen (HBsAg), which is expressed together with unfused HBsAg in genetically engineered yeast cells [95]. In phase I/IIa clinical trials, AS02-based malarial vaccines provided better protection (protecting six out of seven individuals) compared to AS03 and AS04-based formulations in malaria-naïve individuals challenged with *P. falciparum* through bites from mosquitoes [96]. Recombinant AS02-based malaria vaccine has been tested for safety, immunogenicity and efficacy in various phase I clinical trials. Apart from malaria vaccines, AS02 has been tested with tuberculosis, hepatitis B, HIV vaccines and cancer immunotherapy (Table 3). 

**Table 3:** Adjuvant system (AS): Clinical studies.

Adjuvants	Phase	Subjects	Summary	References
AS04/HPV-16/18 (Cervarix^TM^)	III	Girls and women (10–75 years)	Ab responses in serum and cervicovaginal secretions up to 36 months	[97]
	III	>8,000 women (15–25 years)	Vaccine efficacy 92.9% against cervical intraepithelial neoplasia grade 2 and above (CIN2^+^) 34.9 months post vaccination and high vaccine efficacy against CIN2^+^ associated with 12 non-vaccine oncogenic strains (cross-protection)	[98]
	IIIb	HIV-seronegative young African girls (10–25 years)	100% seroprotection, which sustained up to 12-month post-vaccination, high neutralizing Abs and total IgG titers sustained for 8.4 years post-vaccination	[99,100]
	IIIb	Young girls (9–15 years)	Co-administered HPV-16/18/AS04 with inactivated hepatitis A and B (HAB) or dTpa-IPV: immunogenicity was no inferior than any of the vaccine alone	[101]
AS04/HBV (Fendrix^®^)		Elderly, immunocompromised and renal insufficiency patients	Rapid and high seroprotection at 3 month (74% *vs .* 52%) and 3 year (73% *vs .* 52%) compared to HB-alum vaccinated individuals	[102]
AS03 + Influenza A (H1N1 vaccine)	II and III	Volunteers (18–60 years), high-risk elderly (61–88 years) and children (3–9 years)	Higher seroconversion levels and 4-fold Ag-sparing effect compared to nonadjuvanted vaccine, ↑ H1N1 specific CD4^+^ T cells	[103,104,105]
AS03 + Influenza A (H1N1 vaccine)	II	Children (6 months–12 years)	Higher immunogenicity compared to nonadjuvanated vaccines	[106]
AS03 + H1N1 influenza pandemic vaccine 2009		Multiple populations	Vaccine efficacy: 72%–97%	[107]
AS02+RTS,S (Malaria vaccine)	IIb	131 semi-immune adults	Vaccine efficacy: 71% in 9 wks, strong Ab and T-cell responses to CSP	[108]
	IIb	2,022 children (1–4 years)	Vaccine efficacy: 29.9% for first clinical episodes and 57.7% for severe malaria. Vaccine efficacy: 48.6% at 18 months for several malaria cases	[109]
AS02 + Mtb72F (TB vaccine)	I	Purified Protein Derivative (PPD)-negative adults (18–40 years)	↑ Humoral and polyfunctional CD4^+^ T-cell responses expressing CD40L, IL-2, TNF-α and IFN-γ, sustained for 9 months and 1 year after primary and booster immunization, respectively	[110]
AS02 + HBV	III	450 healthy individuals Renal insufficiency patients; inprior HB vaccinated individuals	High seroprotection rates after first (75.9%) and second vaccine doses (99.7%) Seroprotection rate (76.9%) Seroprotection rates were 89.0% one month post-booster vaccination	[111,112]
AS02 + MAGE-A3		Advanced tumor patients, Lung cancer patients	Anti-MAGE-A3-specific Ab responses in 96% of the advanced tumor patients and 30% patients showed ↑ IFN-γ responses	[113,114]
AS01/RTS,S (malaria vaccine)	Preclinical	Rhesus monkeys	↑ Ag-specific Ab and IFN-γ producing Th1-type responses	[115,116]
AS01/MSP-1 (malaria vaccine)	IIa	102 healthy human volunteers	Vaccine efficacy was higher (50%) compared to RTS,S/AS02A vaccine; ↑ CSP-specific IgG titers and polyfunctional CD4^+^ T cells expressing IL-2, IFN-γ, TNF-α, or CD40L	[117]
AS01/M72 (TB vaccine)	I/II	110 PPD-negative human volunteers	Vaccine specific Th1 CD4^+^ T-cell responses compared to M72/AS02 vaccine	[118]
AS01/M72 (TB vaccine)	II	540 children, aged 5–17 months	RTS,S/AS01_E_ induced higher CD4^+^ T-cell responses as compared to RTS,S/AS02D	[119]
AS01/F4 (HIV-1 vaccine)	I/II	180 healthy volunteers (18–40 years)	High frequencies of polyfunctional CD4^+^ T-cell responses characterized by CD40L, IL-2 in combination with TNF-α and/or IFN-γ, which persisted for 44 days post-vaccination.	[120]

### 7.4. AS01

Malaria vaccine adjuvanted with AS02 has shown great promise. However, to further increase the efficacy and to eliminate the infected hepatocytes, the AS01B adjuvant system has been evaluated by GSK. The AS01 adjuvant system is a liposomal formulation comprised of immune-stimulants MPL and QS21 (MPLA+QS21+a liposomal suspension). When tested in rhesus monkeys, RTS,S/AS01 elicited higher antigen-specific antibody and IFN-γ producing Th1-type responses compared to any other adjuvant formulations [116]. Even when tested with MSP-1 antigen, AS01B adjuvanted vaccine elicited the highest anti-MSP1 antibodies and strong Th1 responses characterized by high numbers of IFN-γ secreting cells that were sustained for 24 weeks after final vaccination [121]. A RTS,S/AS01 malaria vaccine was more efficacious than a RTS,S/AS02A vaccine, inducing increased IgG titers and polyfunctional CD4^+^ T cells expressing IL-2, IFN-γ, TNF-α, or CD40L [117] (Table 3). In a more recent clinical trial in children, RTS,S/AS01_E_ also induced higher CD4^+^ T-cell responses as compared to RTS,S/AS02D [119]. 

In view of the strong induction of cell-mediated immunity, AS01B was also evaluated in a human clinical trial with a TB vaccine in comparison to AS02. In this study, M72 antigen was formulated with either AS02 or AS01. While both M72/AS01 and M72/AS02 had an acceptable safety profile and elicited robust polyfunctional M72-specific CD4^+^ T-cell and antibody responses, the highest CD4^+^ T-cell responses were induced by M72/AS01 [118]. Thus, the M72/AS01 was chosen for further vaccine development. 

## 8. Nanoemulsions

Nanoemulsions have a mean droplet diameter of 50–1,000 nm and are particularly suited to mucosal delivery. Although not as extensively studied as liposomes, one nanoemulsion is licensed and several have progressed to human clinical trials. One of the best characterized nanoemulsions is MF59, an oil-in-water emulsion of ~250 nm consisting of the metabolizable oil squalene, Tween-80 and Span 85 (reviewed in [122]). MF59 has an acceptable safety profile and was licensed in 1997 in Europe for an influenza vaccine, thereby becoming the first adjuvant licensed for human applications since alum. MF59 creates a depot effect and directly activates immune cells [123]. 

Two other oil-in-water nanoemulsions, when formulated with smallpox, HIV-1, influenza, or hepatitis B antigens and delivered intranasally, mediated the production of not only robust virus neutralizing antibodies, but also IFN-γ and TNF-α, as well as elevated IgG2a and mucosal IgA levels. These oil-in-water emulsions, designated W_20_5EC and W_80_5EC, consist of 1% cetyl pyridum chloride (CPC), 5% Tween 20 or Tween 80, 8% ethanol in 64% soybean oil and 22% water (NanoBio Corporation, Ann Arbor, MI, USA). When formulated with smallpox, HIV-1, influenza, or hepatitis B antigens and delivered intranasally, these adjuvants mediated the production of not only robust virus neutralizing antibodies, but also IFN-γ and TNF-α, as well as elevated IgG2a and mucosal IgA levels. W_80_5EC was evaluated in a human clinical trial with Fluzone^®^, which demonstrated that this adjuvant is well tolerated. Volunteers received a single intranasal dose with increasing antigen and adjuvant amounts. The highest dose (10 µg) of antigen in combination with 20% W_80_5EC, induced increased serum hemagglutination inhibition antibodies reactive with three influenza strains, as well as antigen-specific IgA in the nasal wash [124].

MF59 has been combined with either a CpG ODN or a TLR4 agonist, E6020, in an influenza soluble trivalent egg-derived antigen formulation. While addition of CpG ODN or E6020 to MF59 did not increase influenza-specific antibody titers, a shift towards a more Th1-biased response was observed which suggests that co-formulation of MF59 with a TLR ligand may tailor the immune response to be more Th1 biased [125]. In contrast, co-delivery of E6020 within MF59 enhanced the magnitude of the antibody levels, as well as increased the breadth of the response, when formulated with Men B antigens or Men ACWY-CRM conjugate vaccine [126]; furthermore, addition of either E6020 or CpG ODN to the Men B vaccine in MF59 increased the percentage of antigen-specific cells secreting IL-2, TNF-α and IFN-γ, again demonstrating a shift towards a Th1 bias. Recently, CpG ODN was shown to both enhance the hemagglutination and virus neutralization titers, and specifically increase the IgG2a levels, when added to a trivalent influenza vaccine (FCC) formulated in MF59. In addition, a much more pronounced Th1 profile, characterized by a potent IFN-γ response, was induced when CpG ODN was added [127]. This effect of addition of the TLR agonists is of particular significance not only for these two vaccines, but also in general when a particular Th bias is needed for protection.

## 9. Conclusions

Many of the vaccines that have been recently developed, or will be in the future, consist of highly pure pathogen-derived antigens. This approach is necessary for vaccine development against live-threatening infectious diseases and cancers. For these antigens to induce robust immune responses, formulation with adjuvants is critical. Adjuvants can enhance the speed and magnitude of the development of immune responses, reduce the amount of antigen and/or vaccinations needed, increase cross-protection and reduce non-responsiveness, in particular of specific populations such as the very young or elderly. In view of these multiple roles, the current trend in vaccine formulation against both cancer and infectious diseases is to include at least one and often multiple components to engage PRRs, and thus activate the innate immune response, as well as at least one compound that stabilizes or targets antigens to the appropriate immune cells. Engagement of PRRs by their ligands is critical for induction of the innate immune response which then directs the adaptive immune response to be either Th1-, Th2- or Th17-biased. This ability to tailor the immune response is particularly important for vaccine development against difficult targets such as chronic bacterial or viral infections and cancer. Several classes of PRRs have been identified, including TLRs, NOD-like receptors (NLR), RIG-I-like receptors (RLRs) and C-type lectin receptors (RLRs). As described in this review, TLR agonists have been most extensively studied as adjuvants ands shown to be very promising. The TLR agonists often act synergistically with other classes of adjuvants used in the formulation or even with other TLR or NLR agonists. In fact, it is now known that one of the most effective vaccines, the live-attenuated yellow fever vaccine, activates multiple DC subsets through TLR2, TLR7/8 and TLR9, which leads to a robust balanced immune response. Aluminum salts and muramyl-dipeptides are two examples of NLR agonists used in licensed vaccines and clinical trials, respectively, and as more agonists of these additional classes of PRRs are identified, we can expect more to be tested as adjuvants.

Currently there are four licensed vaccines that contain multiple TLR agonists, BCG that contains TLR2 and TLR4 agonists, Cervarix and Fendrix that contain alum and MPL, and Cadi-05 (Immuvac) (*Mycobacterium indicus pranii*) against leprosy. This demonstrates that it is possible to overcome any real or perceived disadvantages of multi-adjuvanting. One problem is the increased expense and complexity of manufacturing and formulating a vaccine antigen with multiple adjuvants. Another major issue is the potential for more adverse effects in the recipients; however, the individual components of a combination adjuvant often can be used at a lower concentration than when administered as a stand-alone adjuvant. This may actually reduce side-effects and production costs. A third hurdle is the concern of regulatory agents with novel adjuvants and combinations in particular, which hopefully will become more easily alleviated once more information about the mechanisms of action of adjuvants—and vaccines in general—is gained. For instance, the mechanisms of action have been elucidated for several adjuvants, including MF59, alum, MPL (reviewed in [128]); however, efforts in this area, specifically for combination adjuvants, need to be expanded. Systems biology can now be applied to elucidate molecular signatures to new vaccine candidates in comparison to very effective current vaccines such as the yellow fever vaccine and thus predict efficacy and safety [129]; this is another new area of research that will assist in licensing new vaccine formulations.

In this review we summarized recent and relevant information about many known and new adjuvant combinations including ISCOMs, liposomes, Adjuvant Systems, Montanides and triple combination adjuvants is clear that combinations of adjuvants have multiple advantages, and are critical to achieve the desired degree of vaccine efficacy against many new, challenging disease targets. Furthermore, there is strong evidence supporting inclusion of at least one PRR agonist. As these pathogens target different organs and tissues and cause different types of pathogenesis, there likely will be a need for a panel of combination adjuvants for specific disease targets, routes of delivery and/or populations. As recently described [128], depending on whether mainly humoral or cell-mediated immunity is required to induce protection against a pathogen, a TLR4 or TLR3 agonist might be selected to tailor the response. In addition, the choice of delivery vehicle should depend on the route of immunization, mucosal or parenteral. When targeting specific populations such as neonates or the elderly, adjuvant selection should take the expression levels of PRRs in these populations into consideration. As more information becomes available on the efficacy, safety and mechanism of action, we can expect an increase in the number of licensed vaccines containing various combination adjuvants. 
